# Immunogenicity of a Recombinant Avian Influenza H2 Protein Using an Abdominal Inoculation Model in Chickens

**DOI:** 10.3390/vaccines13090926

**Published:** 2025-08-30

**Authors:** Juan Rondón-Espinoza, Gina Castro-Sanguinetti, Ana Apaza-Chiara, Rosa Gonzalez-Veliz, Alonso Callupe-Leyva, Vikram N. Vakharia, Eliana Icochea, Juan More-Bayona

**Affiliations:** 1 Laboratory of Avian Pathology, Faculty of Veterinary Medicine, Universidad Nacional Mayor de San Marcos, Lima 18092, Peru; jrondone@unmsm.edu.pe (J.R.-E.); gcastros@unmsm.edu.pe (G.C.-S.); ana.apaza2@unmsm.edu.pe (A.A.-C.); rgonzalezv@unmsm.edu.pe (R.G.-V.); josealonso.callupe@unmsm.edu.pe (A.C.-L.); micochead@unmsm.edu.pe (E.I.); 2 Institute of Marine and Environmental Technology, University of Maryland, Baltimore County, Baltimore, MD 21202, USA; vakharia@umbc.edu; 3 Laboratory of Virology, Faculty of Veterinary Medicine, Universidad Nacional Mayor de San Marcos, Lima 18092, Peru

**Keywords:** recombinant H2 protein, intra-abdominal inoculation, cellular immune responses, flow cytometry, antibody levels, cytokine expression, avian influenza

## Abstract

Background/Objectives: Avian influenza represents a major threat to both animal and public health. Our group has tracked avian influenza viruses circulating in wild birds in Peru during the last 20 years. While most of these viruses are low-pathogenic avian influenza strains, some exhibit genetic changes that significantly diverge from common circulating viruses. We selected a highly divergent hemagglutinin H2 gene from a genetically characterized avian influenza virus to develop a recombinant protein using a baculovirus system. Methods: We administered 5 µg and 20 µg doses of the recombinant H2 protein (rH2) into 3-week-old chickens using an abdominal cavity inoculation model to evaluate the activation of innate immune responses. Chickens were euthanized at 24 and 72 h post inoculation and an abdominal lavage was performed to harvest the abdominal cavity content. Results: Infiltrating cells were counted and their cell viability was measured using an Annexin V/PI staining. At 24 h, a large proportion of infiltrating leukocytes were identified as heterophils, monocyte/macrophages and lymphocytes. These proportions changed at 72 h, with a decrease in heterophils and increase in monocyte and lymphocyte pools. We observed strong cellular activity in abdominal leukocytes at 24 h, with a decline in activation levels at 72 h. Cytokine expression suggested a tightly regulated immune response during the 72 h period, while a more sustained response was observed at the 20 µg dose. Antibody levels demonstrated the capacity of the rH2 protein to induce long-term responses. Conclusions: These results revealed that the baculovirus-expressed rH2 protein induces a controlled immune activation, a long-term immune response, holding promise as a potential vaccine candidate for animal health.

## 1. Introduction

Avian influenza is one of the most important viral pathogens affecting a wide range of species including birds and mammals [1,2]. The virus is a single-stranded, segmented and negative-sense RNA virus that encodes multiple structural and non-structural proteins, which are distributed across eight genome segments [3,4]. These proteins include PB2 (polymerase basic 2), PB1 (polymerase basic 1), PA (polymerase acid), HA (hemagglutinin), NA (neuraminidase), M (matrix protein), NP (nucleoprotein), NEP (nuclear export protein), among others [5,6]. Due to genome segmentation and the ability to infect multiple species, avian influenza viruses undergo recombination and reassortment events, leading to the emergence of novel viral forms [7,8,9].

Anseriformes and Charadriiformes are the main wild bird reservoirs for avian influenza viruses [10,11,12]. These migratory wild birds are responsible for the viral dissemination across large geographical areas [13,14]. In Peru, multiple viral subtypes have been identified and characterized over the past 20 years. Our group has implemented a tracking system that has led to the identification of several low-pathogenic avian influenza viruses (LPAIV) in both resident and migratory wild birds along the Peruvian coast. These include H3N8, H4N5, H10N9, H13N2 [15], H2N6, H6N8, H13N6, H6N2 [16], and more recently, the HPAIV H5N1 clade 2.3.4.4b spread in wild and domestic birds in our country [17]. These subtypes are identified as the most commonly circulating viral subtypes in wild birds from the Peruvian coast. Of these, a recently described H2N6 viral isolate appears to be the most divergent from its closest relatives [16]. Thus, in its H2 protein, we identified 21 amino acid substitutions compared to its closest relative isolated from a common Goldeneye-duck (Accession number: QGT49557.1) with only 96.26% genetic identity. H2 viruses are of particular concern, as they were the viral subtype involved in the 1957 pandemic [18,19]. Although circulation of this virus has been replaced by other common influenza viruses [20], the lack of immune exposure in large populations to these H2 viruses leaves them unprotected against the potential emergence of novel H2 viral forms [21]. Therefore, understanding the immune responses against this H2 protein will provide insights into the mechanistic processes of immune activation and further developing of long-term immunity.

Recombinant proteins are commonly used as immunogens in vaccine formulations. For influenza vaccines, subunit alternatives have proven to be highly immunogenic and safe when administered to both humans and animals [22,23]. As a result, multiple subunit vaccines have been approved for use in humans to protect against influenza infections [24,25,26]. Similarly, recombinant vaccines have been widely used to combat common pathogens in both production and companion animals [23]. In addition to their ease, design and manufacturing, these vaccines offer the advantage of allowing discrimination between vaccinated and field-exposed animals.

Following immunization, a wide array of cellular responses is activated to initiate the immune response, which eventually leads to the development of long-term immunity. These immunogen-immune cell interactions can be assessed using a high-resolution approach, such as intra-abdominal inoculation method [27,28]. This strategy allows for the characterization of functional and molecular responses with high robustness using flow cytometry [29]. A multiparametric approach offers an integrative view of immune cell activation, subpopulation characterization, viability, reactive oxygen species (ROS) production, cytokine expression, among others [30,31], as the immune response transitions from acute to adaptive phases through the detection of circulating antibodies. Hence, the objective of our study is to assess the phenotypic features of the cellular innate and long-term immune responses against a recombinant H2 protein using an in vivo chicken model.

## 2. Materials and Methods

### 2.1. Synthesis of H2 Gene

We selected the H2 gene segment of a highly divergent H2N6 virus isolated from a Mallard duck (Accession No. OL355026) identified by our group [16]. The H2 gene was synthesized which contains a BamHI restriction site at the 5′ end, the HA coding region without the transmembrane domain, a 6xHis-Tag at the C-terminal and a Nhel restriction site at the 3′ end. The gene fragment (1.39 kb) was cloned into a pUC57-Amp vector (Biomatik, Kitchener, ON, Canada).

### 2.2. Cloning of H2 Gene into a Baculovirus Vector pAcGP67B

Gene sequence was excised from the plasmid vector using the respective restriction enzymes BamHI and NheI. Simultaneously, the baculovirus transfer vector pAcGP67B (BD Biosciences, Franklin Lakes, NJ, USA) was digested with the same restriction enzymes. The digested vectors were run in 0.8% agarose gel electrophoresis and the DNA bands were excised and purified using the GeneJET Gel Extraction Kit (Thermo Fisher Scientific, Waltham, MA, USA) following the manufacturer’s specifications. The ligation reaction was performed in 10 µL of ligation mix, which contains 2 µL of digested pAcGP67B, 5 µL of H2 gene, 1 µL of ligation buffer, 1 µL (400U) of T4 ligase (New England Biolabs, Ipswich, MA, USA) and 1 µL of nuclease-free water. The mixture was incubated at 4 °C overnight. Competent *E. coli*, clone JM109 (Promega, Madison, WI, USA) cells were transformed with the ligation product (5 µL) and competent cells (3 × 10^5^ CFU/50 µL). The mixture was incubated at 4 °C for 30 min, followed by a heat shock at 42 °C for 60 s in a water bath. The transformed cells were overlaid on ampicillin-containing Luria–Bertani (LB) agar plates and incubated at 37 °C for 20 h. Positive bacterial colonies were screened for the presence of H2 gene.

### 2.3. Production of Recombinant Baculovirus

The plasmid, harboring the H2 gene in pAcGP67B transfer vector, was suspended in nuclease-free water and quantified by spectrophotometry using NanoDrop (Thermo Fisher Scientific, Waltham, MA, USA). This plasmid was mixed with linearized baculovirus DNA (AB Vector, San Diego, CA, USA) and viral transfection was performed using a commercial ProFold™-ER1 system (AB Vector, San Diego, CA, USA), following the instructions provided by the supplier. Briefly, 8 µL of Mirus Bio™ TransIT™-Insect Transfection reagent (Thermo Fisher Scientific, Waltham, MA, USA) was mixed in 1 mL of serum and antibiotic-free insect culture media (S-900), followed by the addition of 2 µL of purified recombinant plasmids and 0.5 µg of baculovirus linearized DNA. Following homogenization, the mixture was incubated for 30 min at room temperature. The mixture was then added in drop-wise manner to an 80% confluent monolayer of *Spodoptera frugiperda* (Sf-9) insect cells. Plates were incubated at 28 °C for 5 h. After this incubation period, one mL of S-900 media was added and incubated for five days. Sf9 cells were harvested for evaluation of recombinant H2 protein production. To generate a large quantity of the recombinant protein for further in vivo assays, we produced the protein in a series of T75 flasks. The recombinant protein was purified using affinity chromatography, taking advantage of the histidine tail (His-Tag) included in the recombinant product. The purified product was then quantified using a fluorometer (Qubit, Thermo Fisher Scientific, Waltham, MA, USA).

### 2.4. Characterization of Recombinant H2 Protein

The H2 recombinant protein was expressed in Sf9 cells. Protein production was evaluated at 24, 48 and 72 h post infection (h.p.i). Protein expression was assessed using two methods, Western blotting approach and imaging flow cytometry. For Western blotting, a sonication step was performed to lyse the cells and release the protein component. The lysate was obtained by centrifugation and mixed with SDS buffer solution. Samples were incubated at 90 °C for 5 min before electrophoresis using a 12% SDS-PAGE Mini protean TGX gel (Bio-Rad, Hercules, CA, USA). The protein gel was transferred to a nitrocellulose membrane and run at 370 mA for 30 min. Immunostaining was performed using an Alkaline Phosphatase (AF) anti-His-Tag monoclonal antibody (1:500 dilution). For flow cytometry, cells were harvested and washed in PBS to remove the media. After washing step, cells were incubated with an Alexa-Fluor 642 anti-His-Tag monoclonal antibody at 4 °C for 20 min, followed by a 30 min incubation at room temperature in the dark. Cells were then washed twice with PBS to remove excess antibody. Following washing steps, samples were fixed using 100 µL of formaldehyde (2%). Ten thousand events were recorded in an imaging flow cytometry (Cytek Biosciences, Fremont, CA, USA). The H2 recombinant protein was quantified using a Qubit fluorometer.

### 2.5. Chickens

Three-week-old broiler chickens were used. All birds were housed in the Experimental Facility of the Laboratory of Avian Pathology at the Faculty of Veterinary Medicine, Universidad Nacional Mayor de San Marcos. This study was carried out in compliance with the guidelines provided by the National Service for Animal Health. The protocol for animal studies was approved by the Ethics committee for animal welfare (Protocol number: CEBA2021-33), of the Faculty of Veterinary Medicine in the Universidad Nacional Mayor de San Marcos. All birds had ad libitum access to feed and water. Chickens were monitored every day for record of clinical signs or any form of distress.

### 2.6. Intra-Abdominal Inoculation

Following recombinant protein purification and quantification, we administered 5 and 20 µg of recombinant protein into the abdominal cavity of 3-week-old broiler chickens to promote the activation of immune responses. The protein was prepared in 100 µL of PBS without calcium and magnesium ^(−/−)^. Injections were made 2 cm to the left of the sagittal line, and 2 cm above the cloacal area. Immune responses were measured at 24 and 72 h.p.i., as representative time points for pro- and anti-inflammatory responses, respectively.

### 2.7. Isolation of Abdominal Leukocytes

Intra-abdominal leukocytes were harvested at 24 and 72 h following administration of recombinant H2 protein. An abdominal lavage was performed after cervical dislocation and exsanguination. Briefly, 20 µL of cold PBS ^(−/−)^ was injected into the lower left quadrant of the abdominal cavity, followed by gentle massage to obtain a homogenized solution. Cells were harvested and maintained at 4 °C. A mock group, consisting of chickens inoculated with PBS, was used as a negative control group, while a heat-killed *E. coli*-stimulated group served as a positive control.

### 2.8. Cell Counts and Viability

The abdominal lavage was recovered in a 50 mL Falcon tube and maintained at 4 °C. Cell numbers were determined using a hemacytometer. Total leukocytes were measured at 24 and 72 h.p.i. For viability determination, Annexin V/propidium iodide (PI) staining was performed using an imaging flow cytometry. In brief, samples were washed twice with Annexin V binding buffer, followed by an incubation with 5 µL of FITC-conjugated Annexin V and 1.0 µL of propidium iodide. Cells were incubated at room temperature for 30 min. Following incubation, cells were washed in Annexin V-binding buffer, followed by a washing step in PBS. Hoechst staining was used as a nuclear marker prior to analysis. Finally, 10,000 events were recorded using an imaging flow cytometer (Cytek Biosciences, Fremont, CA, USA).

### 2.9. Leukocyte Populations

It has been previously demonstrated that the main three leukocyte populations can be identified by flow cytometry using a combinatorial approach based on cellular size, internal complexity and fluorescent monoclonal antibodies for immunophenotyping. We applied this approach to assess the dynamics of these leukocyte populations at 24 and 72 h, following H2 recombinant protein administration. In addition to analyzing cell size (area) and internal complexity (dark field), we used a FITC-conjugated anti-KUL01 antibody to identify the monocyte/macrophage populations. Together, these methods allowed us to determine the cellular dynamics at key time points of acute immune responses. A list of the antibody markers and dyes used is presented in Table 1.

### 2.10. Cellular Activation

The activation of abdominal leukocytes was assessed by measuring reactive oxygen species (ROS) and nitric oxide species (NOS). In brief, abdominal lavages were incubated with a mixture of a mouse anti-KUL01 monoclonal antibody, a nuclear marker (NucBlue live reagent, Thermo Fisher Scientific, Waltham, MA, USA) and 0.5 µL of CellROX (Molecular Probes, Thermo Fisher Scientific, Waltham, MA, USA). Following incubation, the mixture was washed twice in PBS ^(−/−)^. Samples were then fixed in 2% formaldehyde, kept at 4 °C and analyzed by flow cytometry. Similarly, for NOS determination, one million cells were incubated with 0.5 µL of DAF-FM^TM^ (4-amino-5-methylamino-2′,7′-difluororescein) along with the antibody marker and nuclear staining mixture. In both cases, cells were washed in PBS ^(−/−)^ twice to remove the excess staining. Ten thousand events were recorded per sample using an imaging flow cytometer.

### 2.11. Flow Cytometry

Flow cytometry experiments were designed to offer robust data of each parameter analyzed. The assessment started creating a dot plot of individual events (FSC-H vs. FSC-A). Individual events were identified for analysis of events based on size and internal complexity (FSC-A vs. SSC-A, respectively) for preliminary identification of leukocytes, and removal of cellular debris. Out of these events, fluorescent antibodies were included to specifically identify the leukocyte populations and their biological function as indicated in Table 1. In most cases, 10,000 events were recorded.

### 2.12. Gene Expression

Cells were harvested and immediately kept at −80 °C for the analysis of cytokine expression of those markers for pro- and anti-inflammatory profile. Total RNA was extracted using the SV Total RNA extraction kit (Promega, WI, USA) following the manufacturer’s specifications. cDNA was prepared using a Superscript III cDNA synthesis kit (Thermo Fisher Scientific, MA, USA). A real-time qRT-PCR was used to calculate the relative expression of each cytokine using the 2^−ΔΔCt^ Method [32]. Β-actin was used as a housekeeping gene. The qRT-PCR protocol was run using the following parameters: 95 °C per 15 min, followed by 40 cycles of 92 °C per 1 min and 60 °C per 30 s. Melting temperature (Tm) was determined exposing the reaction to temperature increments of 0.3 °C, ranging from 65 °C to 95 °C and registering the fluorescence every 2 s. We used a 7500 Real time PCR system (Applied Biosystems, Waltham, MA, USA). Ct values were registered and used in the calculation method for relative quantification. Primers used in the qPCR protocol are presented in Table 2.

### 2.13. Antibody Detection

Following exposure, the development of a long-term immune response can be evaluated by the detection of serum antibodies. We selected three groups of 2-week-old broiler chickens (n = 6 per group) that were used for evaluation of antibody against H2. A group was inoculated with 20 µg of recombinant protein (group 1), a second group was stimulated with an inactivated H2N6 isolate (group 2) and a third group was inoculated with PBS (group 3). Birds were inoculated intramuscularly at 3 weeks old. Blood samples were taken at 0, 7, 14, 21 and 28 days after inoculation. One hundred microliters of serum were obtained and processed for the detection of antibody levels by HI using a homologous H2N6 viral isolate. Serum (25 µL) was serially diluted in an equal volume of PBS, followed by the addition of 25 µL of H2N6 viral isolate (4 hemagglutinating units, HU). The mixture was incubated for 20 min and red blood cells (RBCs, 0.5%) were added. Following addition of RBCs, the mix was incubated at room temperature for 20 min. Positive reaction was determined based on the appearance of a red dot at the bottom of the well, while the lattice formation at each well indicates a negative reaction.

### 2.14. Statistical Analysis

The statistical analyses were performed using R [33]. We evaluated whether the parameters followed normal distribution using the Kolmogorov–Smirnov tests. For production of recombinant H2 protein, we included three technical replicates (n = 3). For the in vivo assays, we used nine technical replicates (n = 9). We considered six replicates for cytokine expression assays. For all analysis, we used a one-way ANOVA with statistical significance as *p* values < 0.05, and the post hoc analysis of Fisher LSD. Significance levels were annotated as follows: * (*p* value < 0.05) and ** (*p* value < 0.01).

## 3. Results

### 3.1. Modified H2 Gene Segment Inserted into Linearized Baculovirus Genome

**Figure 1:**
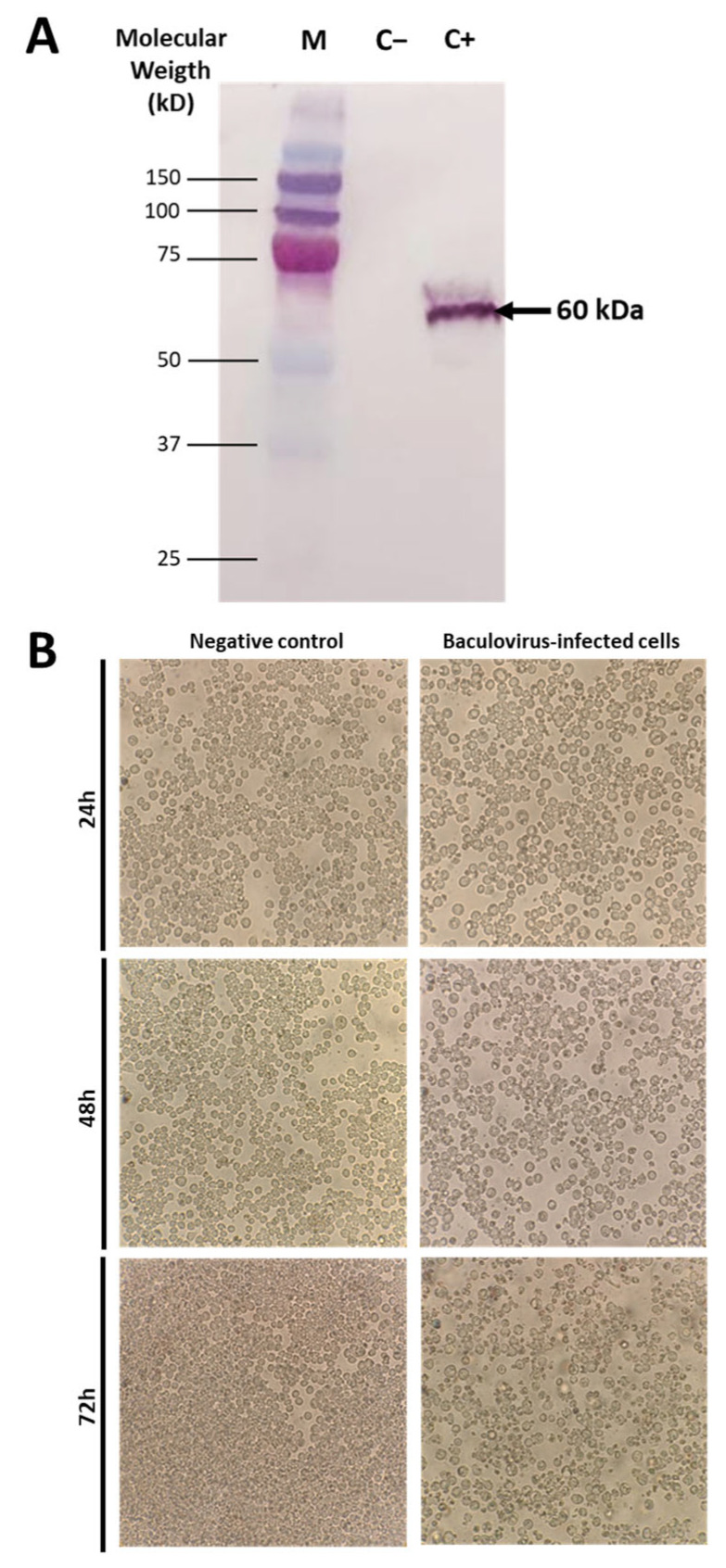
Sf-9 insect cells, infected with the recombinant baculovirus, were harvested at 72 h.p.i. After lysis, cellular proteins were separated on 12.5% SDS-polyacrylamide gel, transferred to a nitrocellulose membrane, and analyzed by Western blotting using a histidine (6xHis) tag monoclonal antibody. M: Pre-stained low molecular weight protein markers from Bio-Rad; C: control Sf-9 cell lysate (uninfected); AIV-H2: recombinant baculovirus infected Sf-9 cell lysate, exhibiting a 60 kDa band of recombinant H2 protein (**A**). Sf-9 cells were mock-infected (negative control) or infected with a recombinant baculovirus for 24, 48 and 72 h and visualized under an inverted microscope. Cytopathic effects were observed in Sf-9 cells infected with recombinant baculovirus as early as 24 h.p.i. (**B**).

Insertion of the H2 gene into a plasmid and viral vector for production of recombinant H2 protein production requires designing the region for stable expression and subsequent purification. After insertion into a standard pUC57 cloning vector, we obtained a 1.39 kb product, detected by agar electrophoresis, corresponding to the expected size of the insert. Further, insertion of this fragment into a baculovirus transfer vector pAcGP67B after transformation into competent *E. coli* was confirmed by agarose electrophoresis, which revealed two bands corresponding to the transfer vector (9.7 kb) and another corresponding to the recombinant H2 protein insert (1.39 kb). A Western blot was run to identify the protein product of a specific size of 60 kDa (Figure 1A). 

### 3.2. Maximum Recombinant H2 Protein Production Was Detected Following 48 h of Inoculation into Sf9 Cells

**Figure 2:**
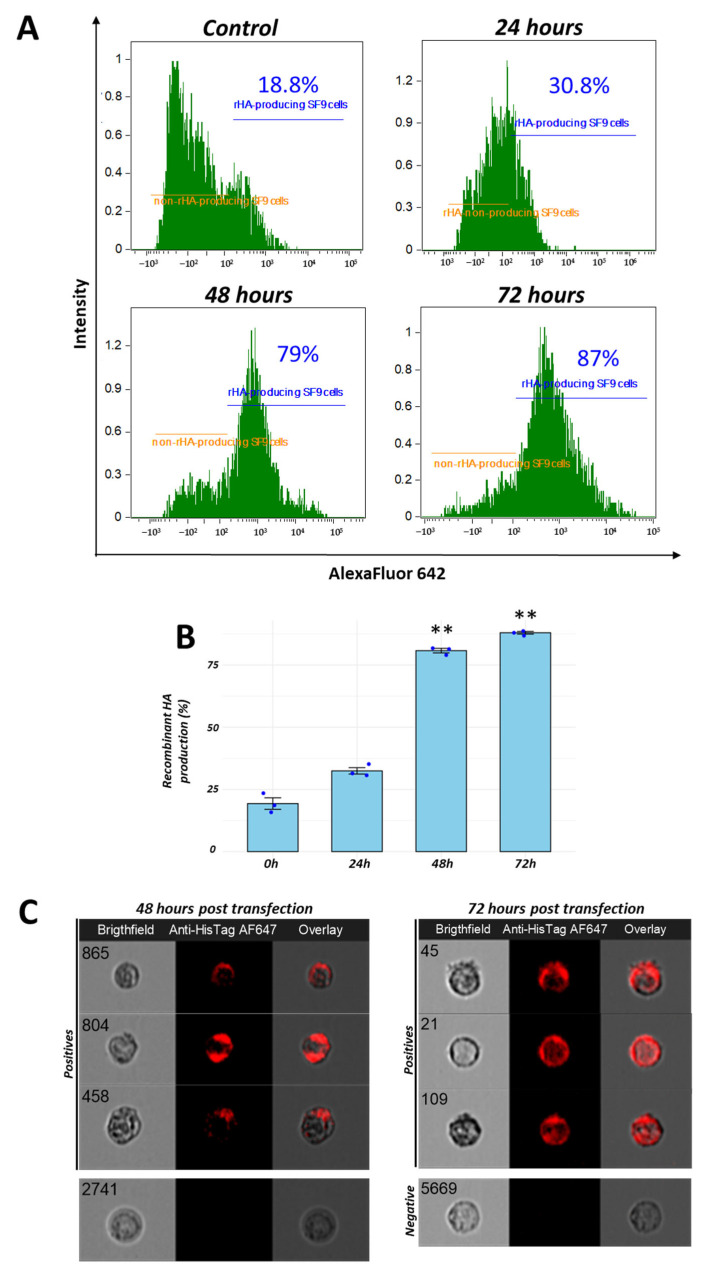
Maximum levels of recombinant H2 production detected between 24 and 72 h by imaging flow cytometry: the expression of recombinant H2 protein was detected at 24, 48 and 72 h following transfection into Sf9 cells, using an AF642 anti-His-Tag monoclonal antibody. An increase in production was observed (**A**). Significant differences in expression were detected at 48 and 72 h following inoculation (**B**). Representative pictures of positive and negative events by imaging flow cytometry are presented in (**C**). ** *p* < 0.01.

Sf9 cells were transfected with a recombinant baculovirus containing the recombinant H2 gene. Normal (negative control) cells did not exhibit any morphological or phenotypical changes (cytopathic effects). However, treated groups showed evidence of changes at 48 h.p.i. (Figure 1B). Cells were then harvested and processed for evaluation by imaging flow cytometry. To assess the dynamics of recombinant H2 protein production, we evaluated Sf9 cells at 0, 24, 48 and 72 h following baculovirus transfection. Cells were processed for flow cytometry staining to determine rH2 expression levels. The expression of recombinant H2 was slightly increased at 24 h (32.5% ± 2.6), significantly higher at 48 h (80.8% ± 1.8), and reached maximum levels at 72 h (88% ± 1.2). Representative histograms for each time point are shown in Figure 2A. A summary graph of the levels at each time point is presented in Figure 2B. A comparison of recombinant protein production at 48 and 72 h revealed a more localized signal at 48 h, while a more dispersed and brighter signal was observed at 72 h in cells analyzed by imaging flow cytometry (Figure 2C). Recombinant H2 protein production was also confirmed by Western blotting, which detected a 60 kDa band as expected. Recombinant protein concentration was quantified by a Qubit fluorometer. Aliquots of 100 µg were stored at −80 °C for further in vivo inoculation assays.

### 3.3. Recombinant H2 Protein Promotes a Leukocyte Infiltration in Chicken In Vivo Infection Model

**Figure 3:**
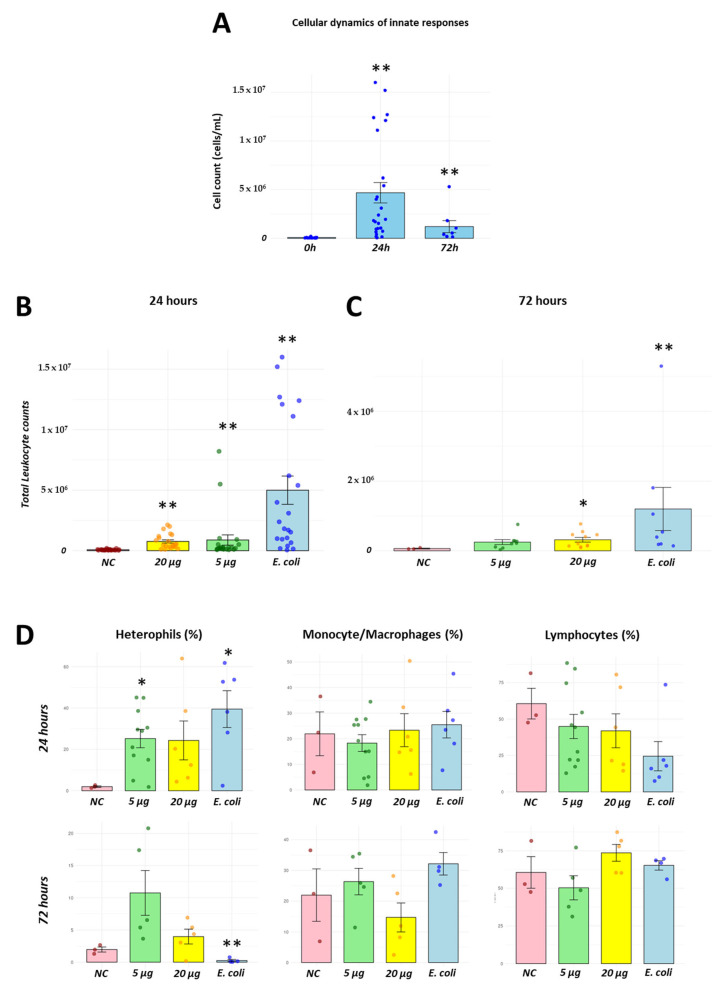
Recombinant H2 protein promotes cellular recruitment into chicken abdominal cavity. Using an intra-abdominal inoculation model, we defined that an inactivated *E. coli* promoted a controlled immune response within a 72 h period (**A**). Inoculation of 5 and 20 µg of recombinant protein induced cellular recruitment after administration into the abdominal cavity and detected at representative time points of pro- (24 h) and anti-inflammatory (72 h) responses (**B**,**C**). Proportion of leukocytes (heterophils, monocyte/macrophages and lymphocytes) were identified using flow cytometry at 24 and 72 h post inoculation (**D**). * *p* < 0.05, ** *p* < 0.01. Negative control (NC), inoculation of recombinant H2 protein at 5 µg (5 µg), inoculation of recombinant H2 protein at 20 µg (20 µg) and inoculation of heat-killed *E. coli* (*E. coli*).

Following recombinant H2 protein purification, we tested two concentrations of recombinant protein: 5 µg and 20 µg. Leukocyte infiltration was assessed at 24 and 72 h as representatives of pro- and anti-inflammatory responses, respectively. Basal levels of leukocytes were 0.34 ± 0.4 × 10^6^ cells. At 24 h, leukocyte infiltration reached to 1.35 ± 0.7 × 10^6^ cells when exposed to 5 µg of recombinant H2 protein, while it reached to 1.04 ± 0.3 × 10^6^ cells when stimulated with 20 µg of the same protein. The heat-killed *E. coli* positive control group showed 5.61 ± 2.4 × 10^6^ total cells (Figure 3B). Cellular infiltration was also measured 72 h post recombinant protein inoculation. At this time point, cell counts were 0.24 ± 0.1 × 10^6^ cells following exposure to 5 µg of recombinant H2 protein, and 0.36 ± 0.03 × 10^6^ cells after exposure to 20 µg of the recombinant protein (Figure 3C). Using the *E. coli* stimulation resulted in a count of 1.13 ± 1.2 × 10^6^ cells. A multiparametric approach was used to analyze leukocyte populations at these time points. Basal levels (PBS-injected, negative control) of heterophils were 1.64% ± 0.46. At 24 h.p.i., heterophils levels increased to 26.47% ± 6.21 when stimulated with 5 µg of the recombinant protein, and 25.99% ± 10.88 after exposure to 20 µg of protein inoculation. Monocyte/macrophage populations did not show significant changes following protein stimulation. Basal levels were 21.26% ± 12.91, while the values for the 5 and 20 µg treatments were 19.49% ± 4.72 and 23.02% ± 8.97, respectively. In terms of lymphocyte levels, a slight reduction was observed following 20 µg of recombinant protein inoculation, with a level of 41.83% ± 13.51. These values were comparable to those observed in the heat-killed group (Figure 3D).

### 3.4. Recombinant H2 Protein Promotes Cellular Recruitment with High Cell Viability

**Figure 4:**
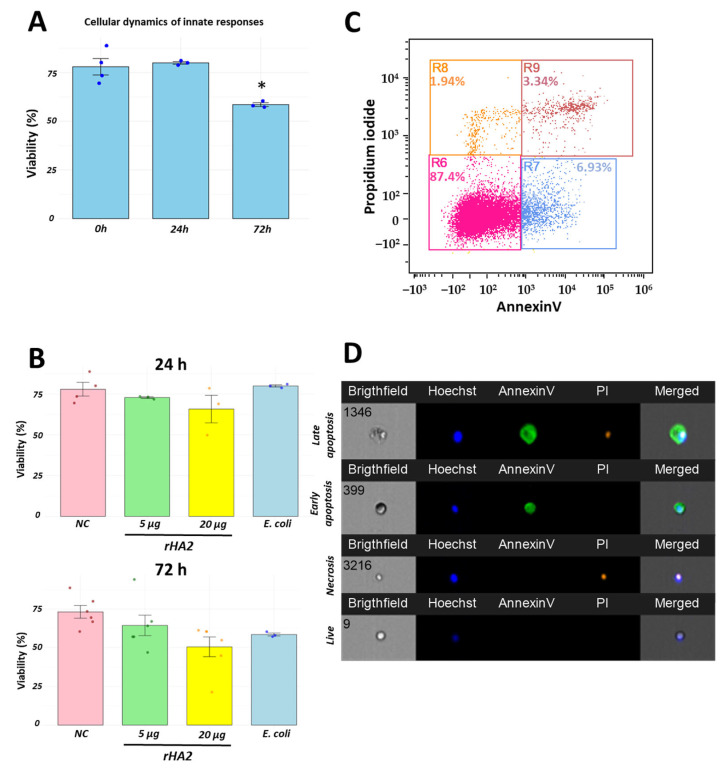
High cellular viability in cellular components recruited by recombinant H2 protein in a chicken abdominal model. Cellular viability decreased gradually as the immune response progresses against a pathogen. Using an *E. coli* model, higher cellular viability was detected at earlier time point while these values were reduced at a later stage of immune response (**A**). Using our recombinant H2 protein, cellular viability was high compared to later state of immune response (**B**). A representative dot plot of flow cytometry is shown in (**C**). Representative events of cellular mortality are presented in (**D**). * *p* < 0.05. Negative control (NC), inoculation of recombinant H2 protein at 5 µg (5 µg), inoculation of recombinant H2 protein at 20 µg (20 µg) and inoculation of heat-killed *E. coli* (*E. coli*).

Intra-abdominal administration of a well-known stimulant, heat-killed *E. coli*, resulted in a depletion of cellular viability at later state of immune response (Figure 4A). To investigate the viability of infiltrated cells following interaction with the recombinant H2 protein, we evaluated the proportions of viable leukocytes using a combinatorial staining of Annexin V and propidium iodide. Overall, proportions of viable remained high across all groups evaluated. Specifically, viability was greater than 75% after 24 h of protein inoculation, with a decrease to over 60% at 72 h. Interestingly, these values were slightly lower when exposed to 20 µg of recombinant protein, approaching those observed in the *E. coli* group (Figure 4B). Representative dot plots of cellular viability and images obtained from imaging flow cytometry are presented in Figure 4C,D.

### 3.5. Recombinant H2 Protein Promotes In Vivo Macrophage Activation

**Figure 5:**
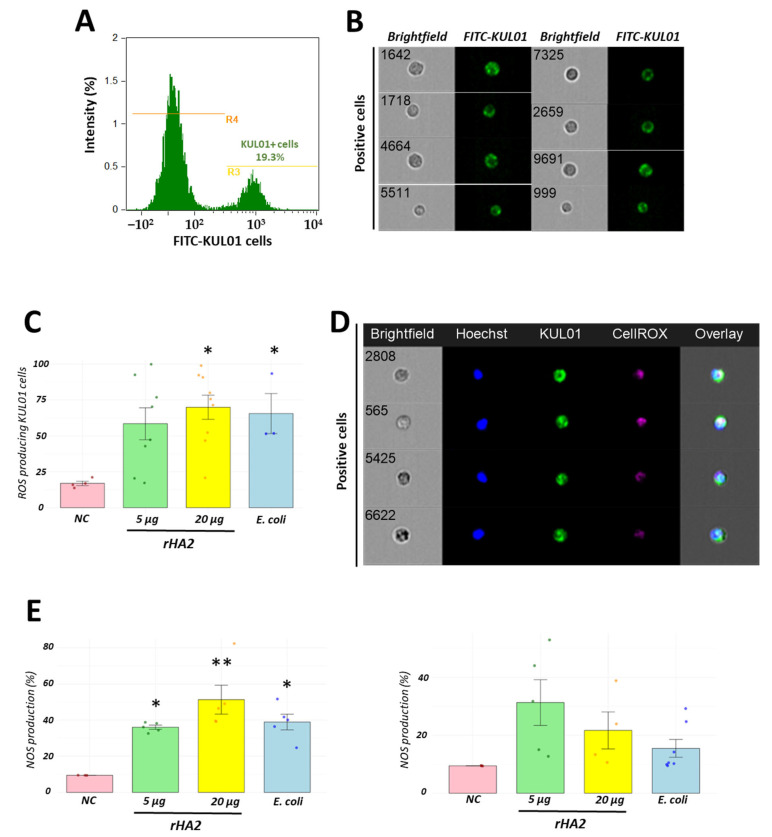
Recombinant H2 protein promotes cellular activation through production of ROS and NO in the recruited chicken leukocytes. Using a FITC-KUL01 monoclonal antibody, we identified the migration rate of the monocyte/macrophage pool into the chicken abdominal cavity following recombinant H2 protein inoculation (**A**). KUL01 positive cells identifying monocyte/macrophages are presented in (**B**). Despite the absence of significant differences following stimulation with 5 or 20 µg of recombinant protein, we observed significant rates of ROS production similar to those observed in the *E. coli* control group (**C**). A multiparametric panel showing ROS-producing KUL01 cells are presented in (**D**). Moreover, DAF-staining was detected at 5 or 20 µg showing the NO production at 24 and 72 h following inoculation (**E**). * *p* < 0.05, ** *p* < 0.01. Negative control (NC), inoculation of recombinant H2 protein at 5 µg (5 µg), inoculation of recombinant H2 protein at 20 µg (20 µg) and inoculation of heat-killed *E. coli* (*E. coli*).

In addition to the monocyte/macrophage differentiation based on morphological features and surface markers, we added a lineage-specific fluorescent monoclonal antibody directed against a surface mannose receptor, such as FITC-KUL01 antibody. Using this method, we identified the proportions of activated cells by measuring ROS production with the CellROX fluorescent dye (Table 1). Basal ROS production in KUL01 positive cells was of 18 ± 3.1%. However, after 24 h of recombinant protein stimulation with 20 µg, ROS levels were significantly higher, similar to those observed in the heat-killed group (positive control). Both the 20-µg recombinant protein and *E. coli* groups had significantly higher ROS production than the negative control group (*p* < 0.05). Further, we analyzed the cellular activation focused on measuring nitric oxide (NO) production. Using the DAF-FM diacetate dye (Table 1), we detected an increase in NO production at 24 and 72 h following recombinant H2 stimulation, similar to those observed in the *E. coli* control group. Basal NO production was 9.44% ± 0.14. At 24 h, NO production increased to 36.84% ± 2.99 and 50.98% ± 20.09, following 5 µg and 20 µg of recombinant protein stimulation, respectively. These levels were significantly different from the control group (* *p* < 0.05, ** *p* < 0.01). At 72 h, NO levels were 31.06% ± 19.57 and 21.9% ± 21.34 for the 5 µg and 20 µg of recombinant protein treatments, respectively. No significant differences were observed between any treatment and control groups at this time point. Together, the ROS and NO production results demonstrated in vivo cellular activation in chickens following recombinant protein stimulation (Figure 5).

### 3.6. A Dynamic Immune Response Was Detected by Cytokine Expression in a Concentration-Dependent Manner

**Figure 6:**
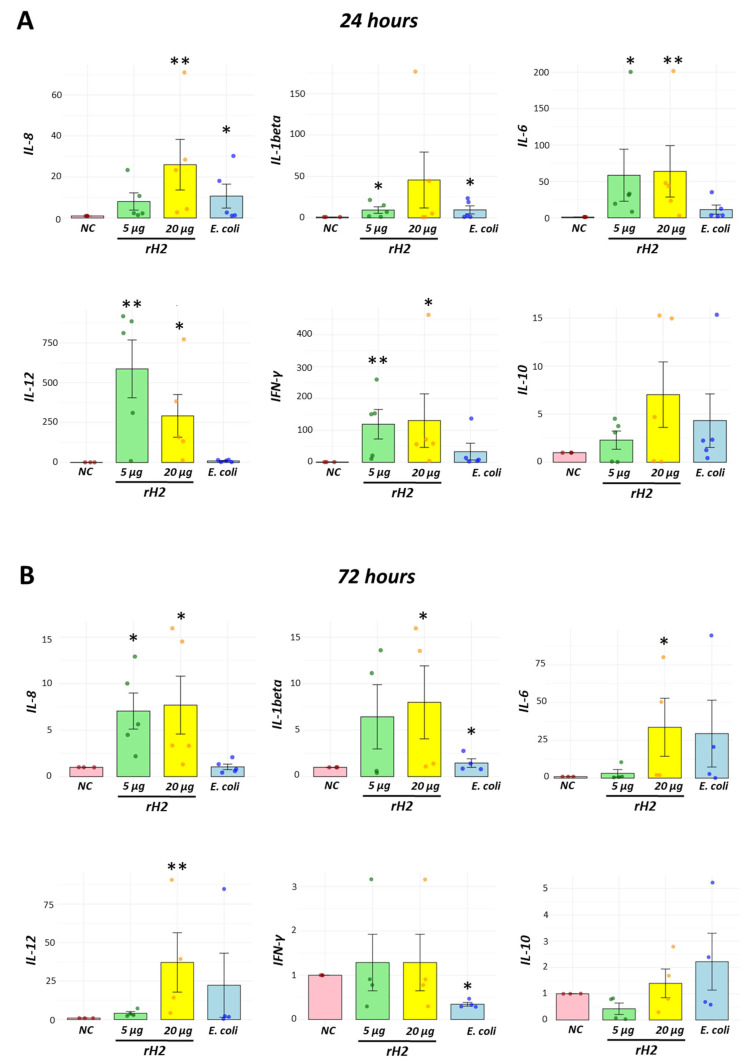
Recombinant H2 protein induces changes in cytokine expression in a concentration-dependent manner. Total RNA was extracted from recruited leukocytes in the abdominal cavity following 24 and 72 h of recombinant H2 protein inoculation. Increased expression of pro-inflammatory (IL-6, IL-12 and IFN-γ) cytokines at 24 h (**A**), whereas reduced levels at 72 h (**B**) define the controlled induction of the immune response. For some cytokines, higher RNA expression in cytokines (IL-1beta, IL-6 and IL-12) was observed in the 20 µg group compared to those in the 5 µg group at 72 h. Negative control (NC), inoculation of recombinant H2 protein at 5 µg (5 µg), inoculation of recombinant H2 protein at 20 µg (20 µg) and inoculation of heat-killed *E. coli* (*E. coli*). * *p* < 0.05, ** *p* < 0.01.

To better understand the response against the recombinant protein, we coupled to the functional assays, the evaluation of cytokine expression using a relative quantification method. Following exposure to the recombinant protein, cells harvested at 24 h expressed significantly higher levels of IL-6, IFN-γ, IL-12 at both concentrations tested. Increased levels in IL-8 expression at 24 h following exposure were detected when birds were stimulated with 20 µg of recombinant protein but not when exposed to 5.0 µg. Conversely, IL-1beta expression was increased when exposed to 5.0 µg of rH2, but no increased levels were detected at 20 µg exposure. No differences in IL-10 expression were detected at 24 h post stimulation (Figure 6A).

After 72 h of recombinant protein stimulation, higher levels of IL-8 expression were detected compared to the negative control group, but reduced levels compared to those at 24 h, when exposed to both 5 and 20 µg of recombinant protein. Increased levels of IL-1beta, IL-6 and IL-12 expression were detected only at 20 µg of protein stimulation. No differences in IFN-γ and IL-10 expression were detected at this time point. Altogether, these results show the dynamics in cytokine expression suggesting the initial step of a resolving phase of immune response against the recombinant H2 protein (Figure 6B).

### 3.7. The Recombinant H2 Protein Promotes the Production of Inhibitory Antibodies

**Figure 7:**
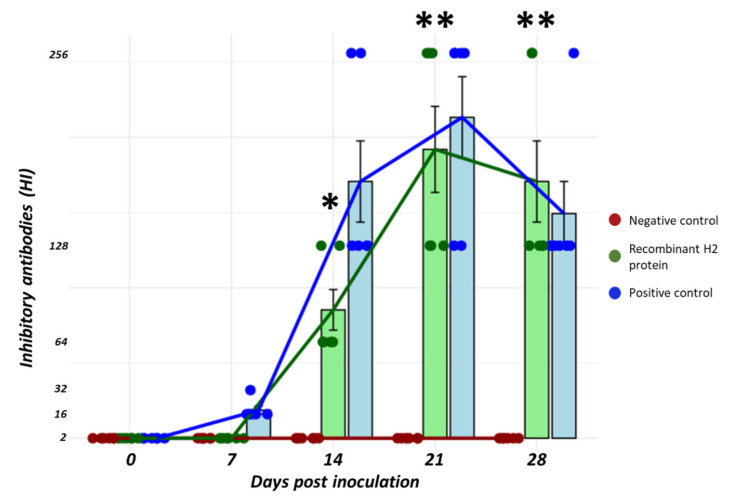
Inhibitory antibodies against a homologous H2N6 isolate in birds stimulated with a recombinant H2 protein. Birds exposed intramuscularly to the recombinant H2 protein (20 µg) induced the production of antibodies at 14 days post inoculation. Earlier dynamics of antibody production were observed in the positive control. Red (negative control), green (recombinant H2 protein) and blue (positive control). * *p* < 0.05, ** *p* < 0.01.

Chickens were exposed to the recombinant protein (20 µg) intramuscularly, and the long-term immunity was assessed by the detection of inhibitory antibodies in serum samples at day 0, 7, 14, 21 and 28 after stimulation. No inhibitory antibodies were detected at day 7 post inoculation (<1:2), while inhibitory antibodies titers ranged from 1:64 (n = 4) and 1:128 (n = 2) at day 14 post inoculation. Antibodies reached the maximum levels at day 21 post inoculation with titers of 1:128 (n = 3) and 1:256 (n = 3). At 28 days, all birds had inhibitory antibody titers of 1:128 (n = 6). Dynamics of inhibitory antibodies had a similar trend in the inactivated virus group, but these antibodies were detected as early as 7 days, with titers of 1:16 (n = 5) and 1:32 (n = 1). Moreover, titers at 14 days post inoculation were significantly higher with the inactivated virus compared to the recombinant protein at 14 days post inoculation (Figure 7).

## 4. Discussion

Avian influenza virus is a major pathogen with significant implications for both animal and public health [34,35]. Its continuously evolving nature has led to the emergence of viral forms with epidemic and pandemic potentials [36,37]. Currently, the highly pathogenic avian influenza virus H5N1 is a major concern, affecting ecosystems worldwide. Although other viral forms, known as low pathogenic, are considered less pathogenic [12], they still contain multiple viral subtypes that can acquire novel characteristics such as mammalian adaptation and increased transmissibility. Thus, it is crucial to pay close attention to those circulating forms alongside other highly pathogenic viruses.

H2-type influenza virus is the viral group that caused the influenza pandemic of 1957 (A/H2N2/1957) [38,39]. While there have been no reports of human infections with this virus in recent years, low-pathogenic H2 viruses continue to circulate among wild bird species [38]. The frequent circulation of these viruses, along with other influenza subtypes such as H5Nx, increases the potential for the emergence of novel, highly pathogenic forms. Indeed, evidence of H2 reassortments events has been observed in our region, including the detection of H2N2 viruses with gene segments derived from both North and South American viruses [40]. For our protein design, we used a viral isolate that carried over 21 amino acid changes in the H2 protein, representing a highly divergent viral form compared to other closely related viruses. Our design aimed to produce the coding protein without the transmembrane region of the H2, which might enhance the development of immune responses targeting the neutralizing regions of H2. This approach facilitates the production of a secreted form of the recombinant protein, which will be less impacted by the purification process. Hence, we detected the protein production in morphologically viable Sf9 cells, even at later stages of baculovirus replication. These results confirm the synthesis of recombinant H2 with higher titers, enabling us to proceed with in vivo testing following protein purification. This recombinant H2 production reached its maximum levels between 48 and 72 h, significantly higher than earlier time points. Despite this, we performed analysis of samples collected at 48 and 72 h so that maximum yield levels are correlated with maximum protein stability.

The chicken abdominal model evidences that our platform is able to identify both the active phase of immune activation and the resolution phase of the immune response using a well-known stimulus. This is consistent with other studies in avian models, which suggest a 72 to 96 h period for the innate immune response following activation [27,31]. Furthermore, this model enables high-resolution assessment of the protein–cell interactions, providing valuable insights into the subcellular events underlying immune activation. Using our recombinant H2 protein, differences in responses of cellular infiltration appeared to be associated in a dose-dependent manner. Although the numbers are shown higher even at 5 and 20 µg of inoculum, these values were slightly higher in the latter dose. These values corresponding to a pro-inflammatory stage (24 h) reveal the capacity of the recombinant protein to promote leukocyte recruitment into an in vivo compartment. At the anti-inflammatory stage (72 h.p.i.), we observed a decrease in the proportion of leukocytes, indicating a resolution of the inflammatory response. However, leukocyte levels at 72 h following stimulation with 20 µg of recombinant protein suggest that an active immune response may still be ongoing at this time, similar to the response observed with well-established stimulants. Conversely, the dynamics of activation retuned to normal at this time point, when stimulated with 5.0 µg of recombinant protein, indicating a shorter immune activation. Altogether, these results revealed that inoculation of recombinant H2 protein effectively activates the immune response that is highly controlled in a time-dependent manner, and dependent on the inoculation dose, particularly during the later stages of immune activation. These results suggest that immune activation using a 20 µg dose may induce a robust immune activation for a sustained long-term immune development.

In poultry, there are well-known immunization strategies for prevention and control of common pathogens. Subcutaneous, intramuscular, spray, eye-drop/intranasal, oral and in ovo vaccinations are implemented on-farm, each showing differential benefits for disease prevention, depending on the specific poultry pathogens involved. Our results indicate that intra-abdominal administration of our recombinant H2 protein promotes activation of both innate and adaptive responses following antigen–cell interaction, events that might be difficult to assess using common immunization methods. However, it is not our intention to propose this route as an alternative immunization strategy for chickens. Using the intra-abdominal inoculation as alternative of immunization in poultry is not suitable as it would require reallocating resources and logistics, potentially causing detrimental effect on overall farm production. Nevertheless, we demonstrate that the abdominal model can be effectively used as a strategy to assess immunogenicity of vaccine candidates in animal models.

Despite the significant cellular migration observed, we assessed whether this immune activation response was accompanied by viable cells for the contribution of phenotypic function of the immune response. This active response is essential for the development of a sustained and highly controlled immune response. A higher cell viability identified at earlier time points compared to the late time point is consistent with the development of immune responses described in other animal models [41]. An anti-inflammatory response is correlated to the development of cellular mortality such as necrosis and apoptosis; thus, it is considered as a transitional phase to the adaptive responses. This is also confirmed with viability results where we expected a large proportion of cell death at later phases of immune responses compared to those in an acute phase.

Monocyte/macrophages are considered one of the first line of defenses against pathogens. Knowing the absence of local monocyte/macrophages in the chicken abdominal cavity, the identification of a large pool of this cellular group further supports an active response against the recombinant H2 protein. Although we did not observe differences in the monocyte/macrophage proportions at both concentrations, levels of cellular activation, in terms of ROS production, were significantly higher when stimulated with 20 µg of recombinant protein, after 24 h of administration. These results demonstrate that the recombinant H2 protein induces cellular responses, specifically at high protein concentration. On the other hand, NO species production is not dependent on the recombinant H2 protein concentration. Nevertheless, differences in ROS and NO production might be related to the dynamics of these byproducts during the cellular activation, where the NO species production is peaked at later phases compared to ROS activation. Altogether, these results demonstrate that the recombinant H2 protein induces early immune responses, which are detectable at the monocyte/macrophage levels, along with cellular mechanisms that are tightly controlled. In addition to its contribution to the first line of defense against a pathogen or immunostimulant, these cells are also a pivotal part of the induction of long-term immune response.

We assessed the cytokine expression at 24 and 72 h post inoculation with recombinant H2 protein. Cytokine profile reveals a pro-inflammatory profile, which occurs at 24 h. However, differential expression was observed in the cytokine analyzed. Analysis at 72 h post inoculation suggests that resolving phase of inflammatory process has occurred when stimulated with 5 µg of recombinant protein, while this is still active when stimulated with a higher concentration. This increased immune process at 72 h (20 µg) may contribute to the development of long-term responses compared to the lower concentration (5 µg). Thus, our results indicate that these coordinated and timely controlled acute responses will lead to an effective activation of adaptive responses. We consider that these results of leukocyte infiltration and cellular activation are key determinants for proper development of long-term responses, specifically at 20 µg of recombinant protein concentration.

Evaluation of long-term immunity was determined by analysis of inhibitory antibodies in serum samples. Our results indicated that the recombinant H2 protein is capable of promoting antibody production with similar dynamics to those using an inactivated H2 virus. However, we detected a slightly slow antibody development in those birds stimulated with the recombinant protein. Furthermore, despite the similar trend in the development of antibody production, slightly lower levels were also found in the recombinant group, mainly at the early stage of development. These results indicate that the recombinant protein is able to induce sustained inhibitory antibodies against a homologous virus. Although we have shown that our recombinant H2 protein is immunogenic at the innate and adaptive immune levels, our results do not provide evidence of protection which is critical for a vaccine candidate. Thus, further studies are needed to define whether the use of adjuvants (types, concentration, etc.), antigen dose, among others, would improve the development of these responses and further offer protection to a live challenge. Nevertheless, our results indicate that the recombinant H2 protein is a promising antigen to be evaluated as a vaccine candidate against influenza H2 infections.

## 5. Conclusions

Altogether, our results indicate that the recombinant H2 protein induces cellular short-and long-term immune responses, which are tightly regulated under an intra-abdominal inoculation chicken model. These results place this recombinant protein as a prospective immunogen to be assessed as a vaccine candidate against H2 influenza infections. Further studies, such as challenge studies, are required to assess whether these responses will confer protection to exposed individuals.

## Figures and Tables

**Table 1:** Antibody and dyes used for flow cytometry analysis.

Monoclonal Antibody/Dyes	Fluorochrome	Manufacturer	Working Volume
Mouse anti-chicken KUL01	FITC	Southern Biotech	0.1 µL
CellRox Deep Red reagent	AF642 Deep red	Life Technologies	0.4 µL
NucBlue live Reagent	Hoechst 33342	Life Technologies	2.5 µL
DAF-FM diacetate	FITC	Molecular Probes	0.1 µL
Annexin V	FITC	BD Biosciences	2.5 µL
Propidium iodide	PI	Thermo Fisher Scientific	0.1 µL

**Table 2:** Primer sequences used for cytokine expression analysis.

Gene	Primer Name	Sequence (5′-3′)
IFN-γ	IFNG-F	TGTAGCTGACGGTGGACCTA
	IFNG-R	GCGGCTTTGACTTGTCAGTG
IL-1beta	IL-1beta-F	GCATCAAGGGCTACAAGCTC
	IL-1beta-R	CAGGCGGTAGAAGATGAAGC
IL-6	IL6-F	CTCCTCGCCAATCTGAAGTC
	IL6-R	CCCTCACGGTCTTCTCCATA
IL-8	IL8-F	CCTCCTCCTGGTTTCAGCTG
	IL8-R	TGGCGTCAGCTTCACATCTT
IL-10	IL10-F	CGCTGTCACCGCTTCTTCA
	IL10-R	TCCCGTTCTCATCCATCTTCTC
IL-12	IL12-F	TATCCCAAGACCTGGAGCAC
	IL12-R	GCCCAGTCTTTGGAATCTGA
TGF-beta	TGFbeta-	GATGGACCCGATGAGTATTGGGC
	TGFbeta-R	GGGACACGTTGAACACGAAGAAG
Β-actin	Bactin-F	CACCACAGCCGAGAGAGAAAT
	Bactin-R	TGACCATCAGGGAGTTCATAGC

## Data Availability

The raw data supporting the conclusions of this article will be made available by the authors on request.

## Abbreviations

The following abbreviations are used in this manuscript:

HA	Hemagglutinin
H2	subtype-2 hemagglutinin
µg	Micrograms
h.p.i.	Hours post inoculation
LPAIV	Low-pathogenic avian influenza virus
ROS	Reactive oxygen species
DNA	Desoxyribonucleic acid
SDS-PAGE	Sodium dodecyl sulfate polyacrylamide gel electrophoresis
PBS	Phosphate-buffered saline
FITC	Fluorescein isothiocyanate
PI	Propidium iodide
NOS	Nitric oxide species
